# Habitat Selection and Intrapopulation Spatial Segregation in an Arctic Top Predator at the Southern Limit of Its Range

**DOI:** 10.1111/gcb.71024

**Published:** 2026-09-05

**Authors:** Tyler R. Ross, Gregory W. Thiemann, Martyn E. Obbard, Kevin R. Middel, Joseph M. Northrup

**Affiliations:** ^1^ Department of Biology York University Toronto Ontario Canada; ^2^ Faculty of Environmental and Urban Change York University Toronto Ontario Canada; ^3^ Wildlife Research and Monitoring Section, Ontario Ministry of Natural Resources, DNA Building Trent University Peterborough Ontario Canada; ^4^ Environmental and Life Sciences Graduate Program Trent University Peterborough Ontario Canada

**Keywords:** habitat selection, intrapopulation variation, polar bear, sea ice, southern Hudson Bay, *Ursus maritimus*, utilization distribution

## Abstract

Accounting for spatial structure within populations is critical for defining appropriate scales of management, particularly in the Arctic, where habitats of ice‐dependent species such as the polar bear ( *Ursus maritimus* ) are being transformed at an accelerated rate. We examined broad‐scale patterns of space use and finer‐scale habitat selection by adult female polar bears in James Bay, located at the southernmost extent of the species' global range. Using satellite telemetry data collected during the annual on‐ice period from 2012 to 2017, we estimated individual‐ and population‐level utilization distributions (UDs) and applied hierarchical clustering to assess whether individuals formed distinct spatial groups based on the degree of UD overlap. We also fit step‐selection functions (SSFs) to evaluate selection for sea‐ice concentration, ocean depth, and distance to coast. Fifteen bears with data spanning at least one complete on‐ice period had individual 95% UDs averaging 74,770 km^2^ (SE = 11,663). Results of hierarchical clustering identified two distinct spatial groups: three “resident” bears remained within southern James Bay, whereas 12 “non‐resident” bears had larger UDs extending into Hudson Bay. Overlap between groups remained near zero throughout winter, including during the breeding season. Step‐selection analyses, which included an additional four bears (*n*_total_ = 19), indicated consistent positive selection for high sea‐ice concentration, with individual variation in selection strength. For non‐resident bears, selection for distance to coast and ocean depth varied seasonally, with bears selecting areas farther from the Ontario coast during freeze‐up, closer to the coast during April–May, and deeper offshore areas during breakup. Together with previous genetic evidence, our results suggest resident southern James Bay bears represent a spatially structured component of the broader subpopulation that warrants continued monitoring and explicit consideration in future harvest‐risk assessments and conservation planning, particularly as sea‐ice loss continues to alter habitat availability.

## Graphical abstract

Located at the southern extent of the species' range, polar bears in James Bay exhibited intrapopulation variation in space use, resulting in two spatial clusters during the on‐ice season. Some bears remained within southern James Bay, whereas others ranged more broadly north of the bay. Despite these differences in broad‐scale movements, individuals showed strong fidelity to James Bay and consistently selected areas of high sea‐ice concentration. Continued sea‐ice decline may force southern‐resident bears either to shift their distribution northward or face regional extirpation, potentially resulting in the loss of genetic diversity and diminished adaptive capacity of this southern sentinel population.

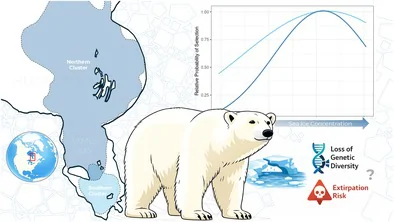

## 1 Introduction

Individuals within a population often exhibit different habitat‐use strategies due to both intrinsic (e.g., genetic) and extrinsic (e.g., local variation in habitat quality) factors, resulting in spatially structured populations characterized by intrapopulation variation in movement patterns, reproduction, and genetics (McCullough 1996; Morris 2003). Hence, although wildlife populations are typically defined as groups of interbreeding individuals that are demographically or spatially disjunct, in practice, their boundaries are often ambiguous and scale‐dependent, as broader populations may encompass smaller units with distinct ecological or genetic characteristics (Wells and Richmond 1995; Camus 2002). Accounting for spatial structure within populations is important for identifying appropriate scales of management, particularly in the context of climate change, which may differentially impact wildlife based on their distribution (Wiens and Bachelet 2010; Sahanatien et al. 2015; Allen and Singh 2016). This is especially relevant in Arctic environments where temperatures have warmed nearly four times faster than the global average, resulting in rapid, regionally distinct declines in sea‐ice habitat (Steiner et al. 2021; Rantanen et al. 2022). Thus, for sea‐ice‐dependent species such as polar bears ( *Ursus maritimus* ), understanding region‐specific patterns of space‐use and habitat selection is essential for predicting future impacts and crafting adaptive conservation strategies that reflect the different spatial extents at which climate change may be affecting the species across its range.

Polar bears are distributed throughout the ice‐covered waters and coastal areas of the circumpolar Arctic where they often exhibit fidelity to large seasonal home ranges (Ferguson et al. 1999; Derocher et al. 2004; Sahanatien et al. 2015). Their movements are closely linked to sea‐ice dynamics, as they preferentially select for areas with high sea‐ice concentration over the continental shelf where ocean productivity and access to prey are greatest (Derocher et al. 2004; Stirling and Derocher 2012). The global population of polar bears is divided into 20 relatively discrete subpopulations based on satellite telemetry, results of mark‐recapture studies, local knowledge, natural barriers, and management considerations (Taylor and Lee 1995; Obbard and Middel 2012; Durner et al. 2018). Despite general patterns in habitat selection, polar bears within the same subpopulation have exhibited divergent space‐use strategies, including differing home range distributions, seasonal use of terrestrial versus sea‐ice habitats, and distinct genetic profiles (Mauritzen et al. 2001, 2002; Peacock et al. 2015; Sahanatien et al. 2015; Pagano et al. 2021; Wilson et al. 2022). In some instances, intrapopulation variation has proven substantial enough to warrant designation of distinct units. For example, polar bears in East and Southeast Greenland, once considered a single population, were recently reclassified as two subpopulations due to differences in movement patterns, habitat selection, and genetic structure (Laidre et al. 2022).

The southern Hudson Bay polar bear subpopulation (SH) occurs in the southernmost continuously occupied area of the species' range, encompassing much of southeastern Hudson Bay and the entirety of James Bay (Jonkel et al. 1976; Kolenosky and Prevett 1983; Obbard and Middel 2012). Situated within the seasonal sea‐ice ecoregion, SH undergoes an annual freeze–thaw cycle during which sea ice forms each fall and melts entirely in the summer, forcing bears ashore for extended periods without access to their primary marine prey (Amstrup et al. 2008; Obbard et al. 2016). Critically, SH experiences one of the longest ice‐free seasons of any polar bear subpopulation, and this period has lengthened by 20–30 days since 1979 (Obbard et al. 2016; Stern and Laidre 2016).

Similar to other subpopulations, management efforts, including administration of harvest quotas, are established and implemented at the subpopulation level based on the presumption that SH represents a single, contiguous demographic unit (Taylor et al. 2001; Obbard and Middel 2012). However, much of our understanding of polar bear space use within SH is based on individuals captured and collared along the Ontario coast of Hudson Bay, located in the western portion of the subpopulation. The limited data available for polar bears in James Bay indicate they exhibit distinct movement patterns (Obbard and Middel 2012; Viengkone et al. 2018; Middel and Obbard 2024). For instance, bears collared in James Bay between 1997 and 2009 (*n* = 4) remained entirely within James Bay for most or all of the on‐ice season, whereas those collared in western SH tended to remain in the northwestern portion of the subpopulation (Obbard and Middel 2012; Middel and Obbard 2024). Genetic analyses of population structure within SH and among adjacent subpopulations identified a distinct genetic cluster comprised of those individuals that remained in southern James Bay, likely reflecting limited spatial overlap during the spring mating season (Crompton et al. 2008; Peacock et al. 2015; Viengkone et al. 2018). However, several bears captured along the western coast of James Bay were assigned to the same genetic cluster as bears in western SH and two neighboring subpopulations, suggesting movement patterns and behavior may vary among individuals within James Bay as well (Viengkone et al. 2016).

The distinctive genetic structure of polar bears in southern James Bay has led to speculation that these bears may constitute a separate demographic unit (Viengkone et al. 2018; Laforest et al. 2018), though these findings are based on limited sample sizes. Consequently, the need for further research in James Bay, particularly focused on polar bear distribution and habitat selection, was identified as a priority (Obbard and Middel 2012), especially since bears at the southern limit of the species' range are thought to be at the highest risk of near‐term climate‐mediated shifts in distribution and population decline (Obbard et al. 2007, 2016; Amstrup et al. 2008; Molnár et al. 2020). Moreover, delineating distinct populations and their associated habitat characteristics is critical for identifying and conserving local adaptations, which underpin the adaptive capacity and long‐term viability of polar bears in SH and beyond. Accordingly, we present the first study focused on polar bear space use and habitat selection in James Bay. Specifically, we estimated individual‐ and population‐level utilization distributions (UDs) and quantified habitat selection during the on‐ice period using movement data collected between 2012 and 2017. Given the distinct genetic profile of polar bears in southern James Bay, we also used hierarchical clustering to assess the degree to which polar bears in James Bay exhibited spatial structuring in their on‐ice space use. Although we expected general habitat‐use patterns to resemble those observed in other subpopulations, polar bears in James Bay may demonstrate localized space‐use strategies, with potentially divergent patterns of distribution and selection that could increase their vulnerability to climate‐mediated habitat change and extirpation.

## 2 Methods

### 2.1 Study Area

**FIGURE 1:**
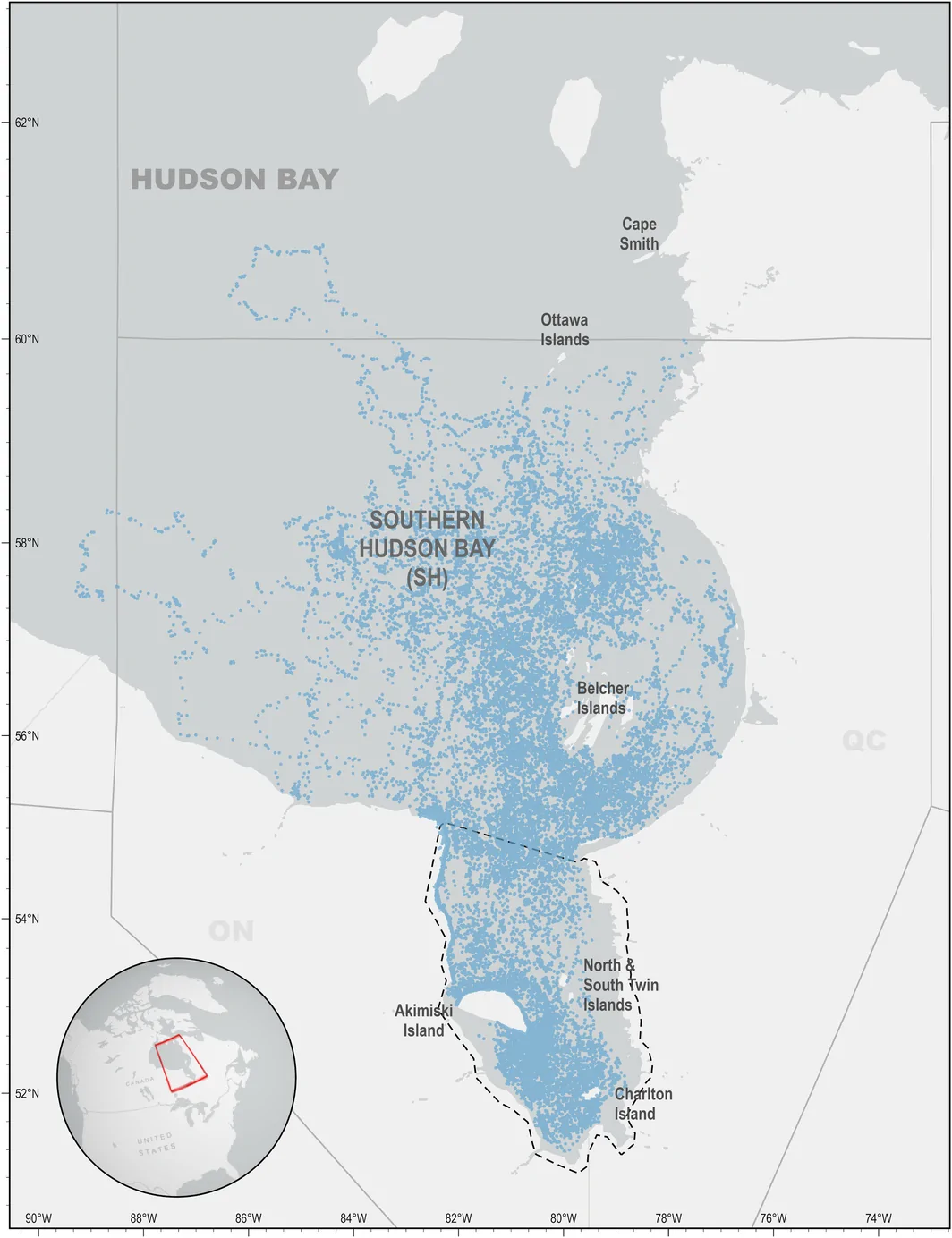
Study area and GPS locations of adult female polar bears in southeastern Hudson Bay and James Bay during the on‐ice period, 2012–2017. Study area (Southern Hudson Bay [SH]), with James Bay outlined by a black dashed line, and location data collected from 19 adult female polar bears equipped with GPS collars between 2012 and 2017 (blue points). Inset map shows the study area in Hudson Bay, Canada (red).

The SH polar bear subpopulation extends over 1 million km^2^ of marine and terrestrial areas, encompassing much of southeastern Hudson Bay and James Bay, as well as inland areas up to 120 km from the coastlines of Ontario and Quebec (figure 1; Kolenosky and Prevett 1983, Obbard and Walton 2004; Obbard and Middel 2012). James Bay extends approximately 430 km southeast from Hudson Bay, with an average depth of 60 m and contains numerous islands, including Akimiski Island and North and South Twin Islands, hereafter referred to as the Twin Islands. Often among the last areas to become ice covered, freeze‐up in James Bay generally begins in November or December (Gagnon and Gough 2005; Andrews et al. 2018; Gupta et al. 2022). Sea ice reaches its maximum concentration around mid‐March and breakup begins in May, with remnant ice occasionally persisting into July (Jonkel et al. 1976; Gupta et al. 2022). Apart from pregnant and post‐parturient females, which stay on land until transitioning to the ice with their cubs in February or March, polar bears in James Bay spend the entire ice‐covered period on the sea ice. They come ashore when the ice begins to melt in the summer, and remain on land until the ice reforms in the late fall (Jonkel et al. 1976; Kolenosky and Prevett 1983; Obbard and Middel 2012; Obbard et al. 2018).

### 2.2 Polar Bear Capture and Data Handling

Polar bears were located on Akimiski Island and along the western coast of James Bay using a Eurocopter EC130 helicopter between September and October 2012–2015. Bears of all age and sex classes, except cubs‐of‐the‐year, were chemically immobilized via remote injection using xylazine (Cervizine 300) and zolazepam‐tiletamine (Telazol) or a combination of medetomidine and zolazepam‐tiletamine (Cattet et al. 1997, 2003). Dependent offspring < 1 year were immobilized with the same drug combinations using a pole syringe. A sample of adult (≥ 5 years) female bears was fitted with Argos‐ or Iridium‐linked satellite collars (Telonics Inc., Mesa, Arizona, USA) programmed to record a GPS location every 4 h. Each collar was equipped with a timed‐release mechanism programmed to drop off after 2 years. Following data collection, all locations recorded on land were excluded since our primary objective was to analyze on‐ice movements and habitat selection.

Research protocols were reviewed and approved annually by the animal care committees of the Ontario Ministry of Natural Resources and Forestry and York University and followed the guidelines of the Canadian Council on Animal Care (http://www.ccac.ca) and the American Society of Mammalogists for the use of wild mammals in research (Sikes 2016).

### 2.3 Kernel Density Estimation and Spatial Clustering

We estimated individual‐ and population‐level utilization distributions for James Bay polar bears during the on‐ice period using 95% kernel density estimation with bandwidths derived using the plug‐in method implemented in the *ks* package (Duong 2020) for the R environment for statistical computing (R Core Team 2023). To reduce autocorrelation among locations, we subset our data by randomly selecting a single location per individual per day. To ensure representative coverage of on‐ice movements, we excluded bears without data spanning at least one complete on‐ice period. Individual utilization distributions were estimated using the full set of daily locations retained for each individual, pooling across consecutive years for those bears tracked over multiple years. Although pooling data may obscure annual variation in movements, we did so to characterize longer‐term individual‐ and population‐level space‐use strategies, including those related to potential changes in reproductive status over time (Schooley 1994; Obbard and Middel 2012). We attempted to quantify individual‐ and population‐level home ranges using autocorrelated kernel density estimation (AKDE), implemented in the *ctmm* package; however, several individuals violated the foundational assumption of range residency (Calabrese et al. 2016; Silva et al. 2022). Among those bears that did meet range residency requirements, several exhibited low effective sample sizes, limiting the reliability of resulting estimates. Therefore, we opted to use the plug‐in method, which also aligns with previous studies of polar bear home range behavior in SH and elsewhere, enabling more direct comparisons with existing findings (Obbard and Middel 2012; Viengkone et al. 2018; Pagano et al. 2021; Jodouin et al. 2025).

Following kernel density estimation, we then quantified pairwise spatial similarity among individuals using the Bhattacharyya coefficient (BC), also known as Bhattacharyya's affinity, which is a symmetric measure of overlap between two probability distributions ranging from 0 to 1, where BC = 0 when distributions share no support and BC = 1 when distributions are identical (Bhattacharyya 1943; Fieberg and Kochanny 2005). To identify possible spatial structure among groups of individuals within James Bay, we applied hierarchical agglomerative clustering using Ward's D criterion, implemented using the *hclust* function in R (R Core Team 2023). Because this method requires a matrix of distances between observations, we first transformed the matrix of pairwise BC values into a dissimilarity matrix by computing Bhattacharyya distances (BD), where $\mathrm{BD}=-\log\left(\mathrm{BC}\right)$ (Bhattacharyya 1946). Using this formulation, BD = 0 for identical distributions and approaches infinity as distributions become increasingly dissimilar. Clustering was then performed to assess whether bears could be grouped according to shared space‐use patterns. To determine the optimal number of clusters, we calculated the mean silhouette width for candidate groupings ranging from 2 to *n‐1*, where *n* is the number of individuals. Silhouette values, which range from −1 to 1, where high positive values indicate observations are well‐clustered and negative values indicate individuals are assigned to the wrong cluster, were computed using the *cluster* package (Rousseeuw 1987; Maechler et al. 2024). The cluster assignment with the highest mean silhouette width was selected, reflecting the optimal balance between within‐cluster similarity and between‐cluster separation (Rousseeuw 1987). Following cluster identification, we estimated population‐level utilization distributions for each group by pooling data across individuals and years. We also estimated seasonal population‐level utilization distributions for each cluster, corresponding to early‐winter (19 December—28/29 February) and late‐winter (1 March—30 June). Transitional periods during the fall and summer corresponding with sea ice freeze‐up and breakup, respectively, were excluded from analyses, as polar bears were migrating onto or off the ice, precluding estimation of stable utilization distributions. Lastly, we compared seasonal utilization distributions between clusters using BC to assess spatial overlap and potential segregation during the winter months and on‐ice breeding season.

### 2.4 On‐Ice Habitat Selection

In addition to examining home‐range‐scale patterns of selection, we assessed the on‐ice habitat selection of polar bears between 2012 and 2017. Specifically, we used step selection functions (SSFs; Forester et al. 2009) to quantify the influence of finer‐scale environmental factors on bear movements. We used the *amt* package (Signer et al. 2019) for the R environment for statistical computing (R Core Team 2023) to regularize polar bear trajectories to near‐uniform fix intervals of 24 h. Location data were segmented into separate bouts when gaps between successive locations exceeded 25 h. Bouts with fewer than three locations—the minimum required to calculate a turn angle—were discarded (Signer et al. 2019). For SSFs, the number of available steps for each used step must be large enough to adequately approximate the underlying spatial point process; however, there is no way to know the appropriate availability sample a priori (Aarts et al. 2012; Johnson et al. 2013; Northrup et al. 2013). Accordingly, we generated three datasets, which included 100, 250, or 500 random steps for each observed step by drawing step lengths from a gamma distribution and turn angles from a von Mises distribution, both fit to the empirical data. Final inferences were based on the dataset for which coefficient estimates stabilized, suggesting convergence and adequate sampling intensity to support robust parameter estimation (Northrup et al. 2013).

We estimated selection using a suite of environmental covariates hypothesized to be important predictors of polar bear habitat selection based on results from previous studies (Durner et al. 2009; Wilson et al. 2016, 2022; McCall et al. 2016). Specifically, we included: ocean depth (ETOPO1 1 Arc‐Minute Global Relief Model; Amante and Eakins 2009), transformed so that depth was expressed as a positive value relative to 0; distance to the nearest coastline of Ontario, Akimiski Island or the Twin Islands; sea‐ice concentration (AMSR2; Meier et al. 2018); and the standard deviation of sea‐ice concentration within a 20 km radius of each used and available point (the approximate mean distance a polar bear moved in 24 h; Wilson et al. 2016). Covariate values were extracted at the end of observed and random steps along the entire movement path of each bear (Signer et al. 2019).

We fit step selection functions using data from individual polar bears pooled across years using a generalized‐additive‐model‐based approach using *mgcv*, which allows for estimation of population‐level selection coefficients, individual‐level random effects, and smooth terms that can account for temporal variation in selection (Klappstein, Michelot, et al. 2024). Prior to analyses, all continuous covariates were standardized and evaluated for pairwise correlation and variance inflation factors to assess collinearity and lack of independence. Models included terms for step length (*step*), log‐transformed step length (*log.step*), and the cosine of turn angle (*cos.turn*), allowing for joint estimation of movement and habitat‐selection parameters (Forester et al. 2009; Avgar et al. 2016; Klappstein, Michelot, et al. 2024). Environmental covariates included sea‐ice concentration (*conc*), local variation in sea‐ice concentration (*conc.sd*), ocean depth (*depth*), and distance to the nearest coastline of Ontario, Akimiski Island, or the Twin Islands (*dist*). Considering results of hierarchical agglomerative clustering indicated bears collared in James Bay belonged to two distinct spatial groups, we included group‐specific terms to evaluate whether selection differed between clusters. We also incorporated estimation of generalized functional responses to sea‐ice availability, recognizing that selection for a particular resource may vary with its relative availability on the landscape or seascape, a phenomenon documented in several species, including polar bears (Mysterud and Ims 1998; Mauritzen et al. 2001; Matthiopoulos et al. 2011). For each stratum, sea‐ice availability was defined as the mean sea‐ice concentration among available locations and was interacted with sea‐ice concentration (*conc × mean.avail.conc*) at used and available locations to estimate whether selection changed as a function of availability (Wilson et al. 2016). To account for possible temporal and individual variation in selection, we adopted a two‐stage modeling approach, fitting a series of candidate models that retained the same core movement and environmental covariates but differed in whether the selection coefficients were allowed to vary over the on‐ice period using smooth terms, and/or among individuals using random effects. We began by fitting a constrained global model (Table S1) using the “double‐penalty” approach, a form of regularization that allows weakly supported smooth and random‐effect terms to be penalized toward zero, effectively removing them from the model (Marra and Wood 2011; Klappstein, Michelot, et al. 2024). Temporal and individual‐level terms reduced to near‐zero were not retained in subsequent models. We then fit a reduced candidate set of biologically plausible models that differed in fixed‐effect structure while retaining the temporal and individual‐level terms supported by the constrained global model. Candidate models were ranked using corrected conditional AIC, and inference was based on the most parsimonious top‐ranking model (Burnham and Anderson 2002; Wood et al. 2016; Klappstein, Michelot, et al. 2024).

Conventional model evaluation and goodness‐of‐fit diagnostics are not directly applicable to SSFs because these models are fit to analyst‐defined matched use‐available data and are commonly estimated using likelihood‐equivalent or numerical‐integration approaches that do not yield a conventional residual structure for assessing model fit (see Fieberg et al. 2018, 2024; Klappstein, Michelot, et al. 2024). Therefore, we evaluated model performance using complementary approaches where possible, including used‐habitat calibration plots (Fieberg et al. 2018) and repeated hold‐out cross‐validation, in which the top‐ranked model was fit 50 times to random subsets comprising 80% of choice sets and evaluated on the withheld 20% by comparing the ranks of predicted choice probabilities for used locations relative to available locations (Forester et al. 2009; Wilson et al. 2022; Klappstein et al. 2022). All analyses were completed in R (R Core Team 2023).

## 3 Results

**FIGURE 2:**
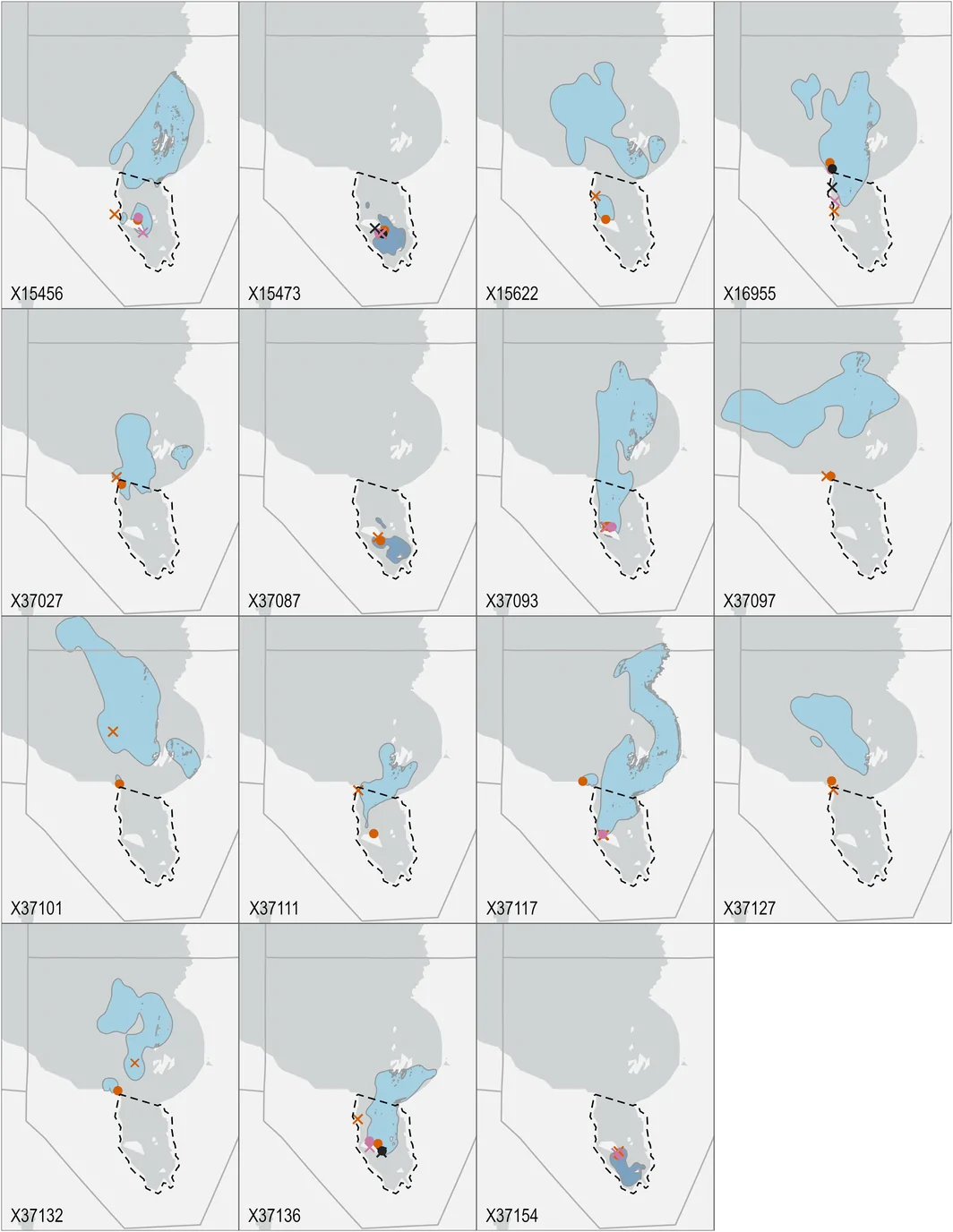
Individual utilization distributions and seasonal sea‐ice transition locations of adult female polar bears in southeastern Hudson Bay and James Bay, 2012–2017. Individual 95% kernel density estimate (KDE) utilization distributions (UDs) for 15 adult female polar bears collared in James Bay between 2012 and 2015. Symbols show seasonal transition locations, with points indicating where bears first moved onto the sea ice during fall freeze‐up and x‐symbols indicating where bears moved off the ice during breakup the following spring (The last GPS locations for bears X37101 and X37132 occurred on the sea ice on 10 August 2015 and 27 July 2014, respectively). Symbol colors denote different seasons for individuals with multiple years of GPS location data. The dark gray polygon denotes the Southern Hudson Bay (SH) subpopulation boundary, and the dashed black line outlines James Bay.

Between 2012 and 2015, we deployed satellite telemetry collars on 19 adult female polar bears and obtained 26,102 on‐ice locations (Figure 1). Bears without at least one full year of data were omitted (*n* = 4), leaving 15 individuals that were included in our analyses of individual‐ and population‐level utilization distributions. After subsetting the data to include only a single location per day, the number of locations per bear ranged from 128 to 653 (SE: 44). Individual utilization distributions varied both in size and geographic extent, with an overall mean of 74,770 km^2^ (SE: 11,663 km^2^; Figure 2). Bear X37087 had the smallest utilization distribution (95% KDE: 13,553 km^2^) and remained almost entirely within *ca*. 140 km east‐southeast of Akimiski Island at the southern extent of James Bay. Bear X37117 had the largest utilization distribution (95% KDE: 151,389 km^2^), which extended from Akimiski Island to the northwestern coast of Quebec near Cape Smith. The remaining utilization distributions were centered near or between Akimiski Island and the Belcher Islands.

**FIGURE 3:**
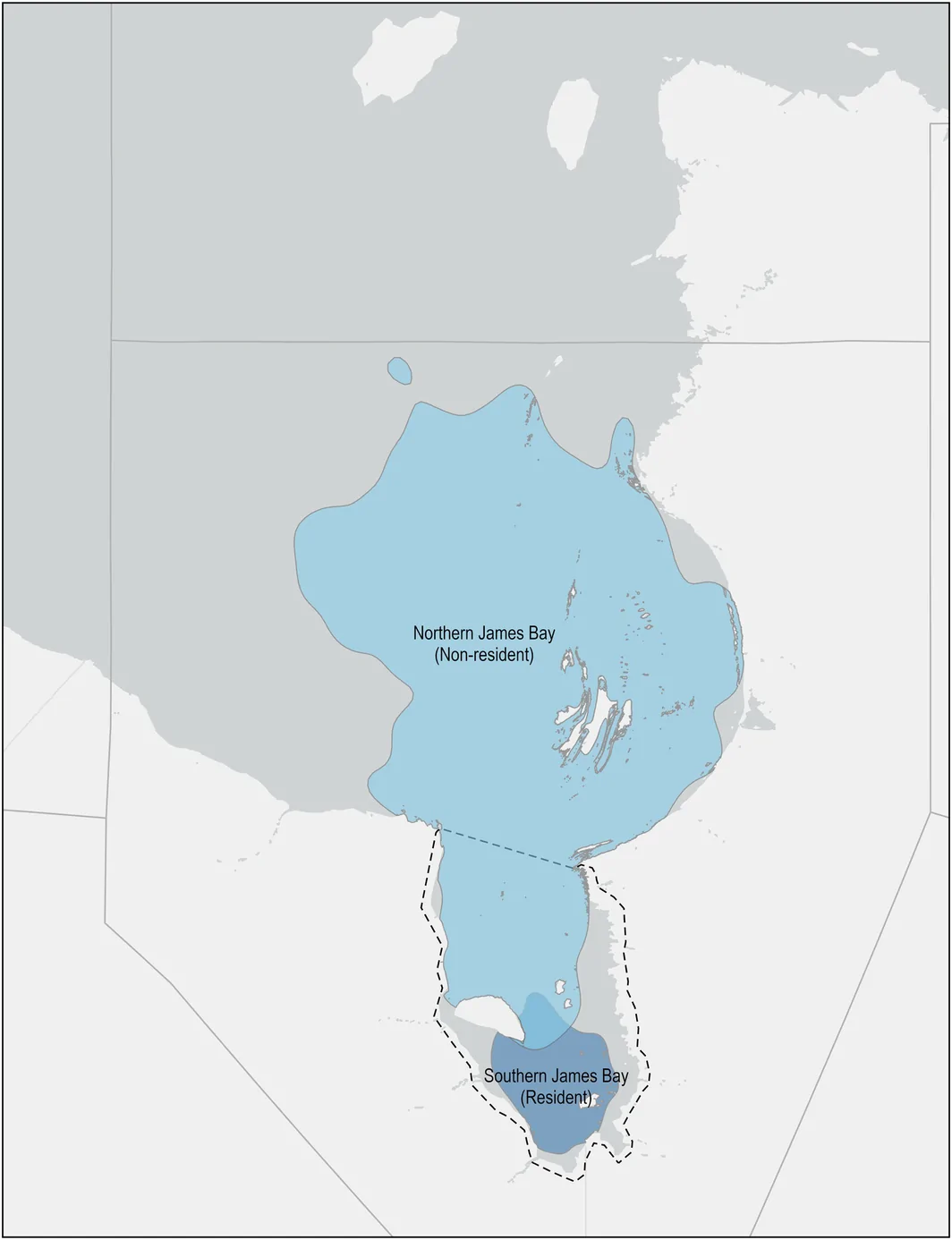
Population‐level utilization distributions of resident and non‐resident adult female polar bears in southeastern Hudson Bay and James Bay during the on‐ice period, 2012–2017. Population‐level 95% kernel density estimates for the northern James Bay cluster (non‐resident bears; light blue) and the southern James Bay cluster (resident bears; dark blue) delineated using hierarchical agglomerative clustering to group individual polar bears according to similar space‐use patterns during the on‐ice season. The dark gray polygon denotes the Southern Hudson Bay (SH) subpopulation boundary, and the dashed black line outlines James Bay.

Hierarchical agglomerative clustering partitioned individuals into groups based on pairwise BD values reflecting similar space‐use patterns (Figure S1). Mean silhouette widths ranged from 0.06 (*k* = 14) to 0.86 (*k* = 2), with the highest value suggesting bears were clustered into two spatially distinct groups: three individuals remained entirely within southern James Bay (hereafter referred to as “resident bears”), whereas the remaining 12 bears (hereafter referred to as “non‐resident bears”) spent the majority of the on‐ice period north of James Bay, near the Belcher Islands (Figure 3). The estimated population‐level utilization distribution of resident bears was 15,323 km^2^, compared to 89,632 km^2^ for non‐resident bears. Population‐level utilization distributions of resident and non‐resident bears remained largely spatially segregated throughout the winter, with a BC value of 0.021 during early winter, increasing slightly to 0.086 during late winter.

All 19 adult female polar bears were included in our analyses of on‐ice habitat selection. After resampling individual tracks to standardized 24 h intervals and removing movement bouts with < 3 locations, we retained a total of 2914 used locations for assessing habitat selection patterns. We fit a total of nine candidate models that included the same movement variables but differed in fixed‐effect environmental covariates, while retaining the temporal and individual‐level terms supported by the constrained global model (Table S1). Sea‐ice concentration and local variation in sea‐ice concentration were highly correlated, so these covariates were not included in the same candidate models. Similarly, ocean depth and distance to the coast were highly correlated among resident bears; therefore, models that included cluster‐specific selection effects considered these covariates separately. The number of available locations did not appreciably affect coefficient estimates; however, values were more consistent among seasonal models that included 250–500 random points. We therefore based subsequent inferences on models that included 500 random points per used location, as estimates appeared to converge to stable values and computational limitations precluded testing of larger available samples. The two top‐ranked models received similar support (ΔAIC = 0.59; Table S1). We selected the highest‐ranked model for inference because it was more parsimonious, as allowing the effect of sea‐ice concentration to differ between resident and non‐resident bears did not meaningfully improve model support. The top‐ranked model included fixed effects for sea‐ice concentration, non‐resident distance to the coast, resident and non‐resident ocean depth, and the sea‐ice functional response. The model also included temporally varying effects for non‐resident distance to the coast and ocean depth, as well as individual‐level random slopes for sea‐ice concentration and non‐resident ocean depth. Variance inflation factors for habitat covariates retained in the final model were low (≤ 1.15), indicating little evidence of multicollinearity among fixed‐effect habitat predictors. Used‐habitat calibration plots indicated that observed distributions at used locations were consistent with those expected based on the fitted model, suggesting adequate calibration across the three environmental covariates included in the final model (Figure S5).

**FIGURE 4:**
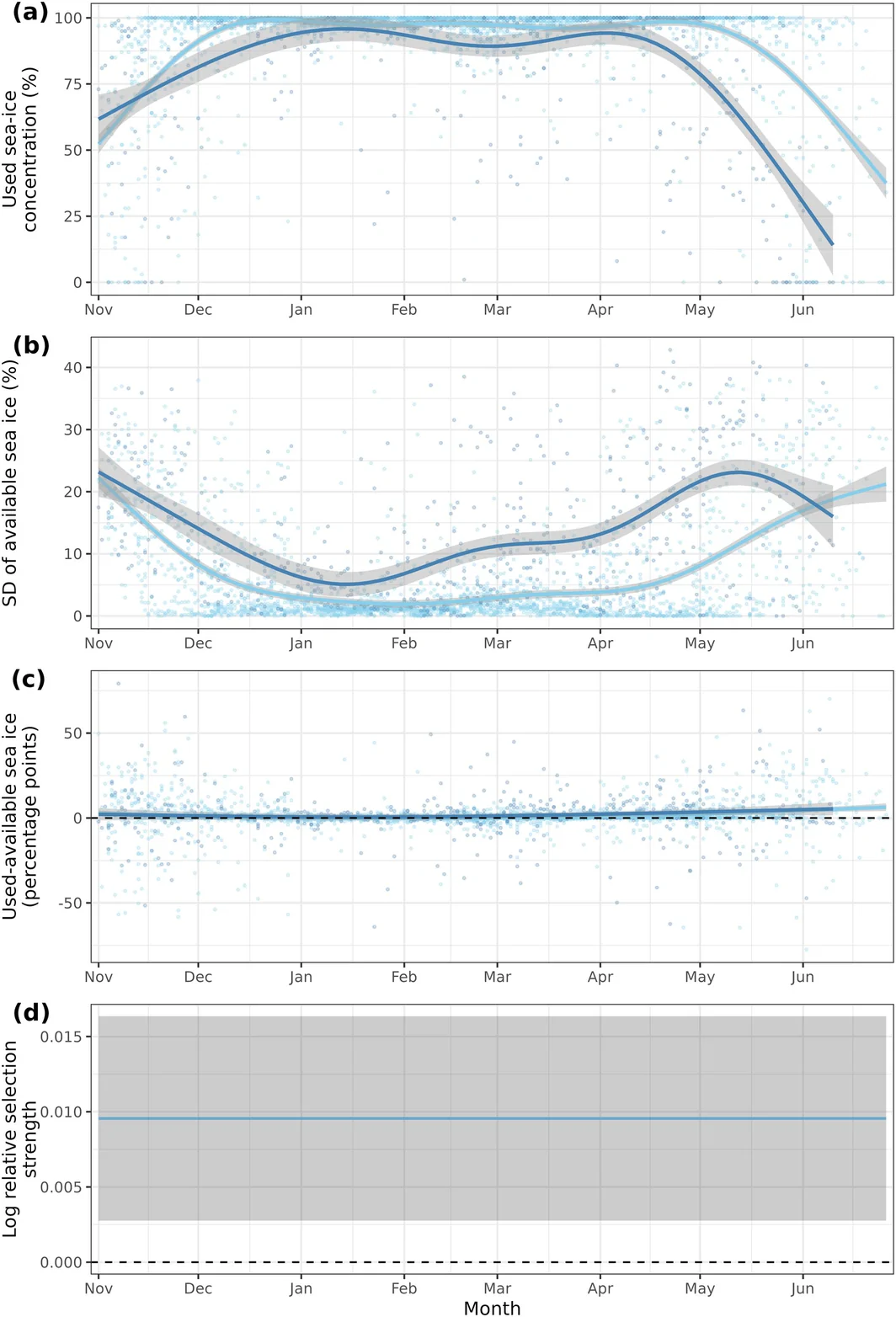
Seasonal patterns in sea‐ice use, availability, and selection by polar bears in southeastern Hudson Bay and James Bay. (a) Sea‐ice concentration at used locations. (b) Within‐stratum variation in available sea‐ice concentration, measured as the standard deviation among available locations in each used‐available choice set. (c) Difference between used and available sea‐ice concentration, calculated as used minus mean available concentration within each stratum. Positive values indicate use of higher‐concentration ice than was locally available. (d) Model‐estimated log relative selection strength, log(RSS), for a 1 percentage‐point increase in sea‐ice concentration; values above zero indicate positive selection for higher sea‐ice concentration. Points in panels a–c show observed values and lines show generalized additive smooths with 95% confidence intervals. Based on results of model selection, the top‐ranking model suggested selection for sea‐ice concentration did not differ between resident and non‐resident bears, nor did it change over time, so (d) shows the combined fixed‐effect log(RSS).

**FIGURE 5:**
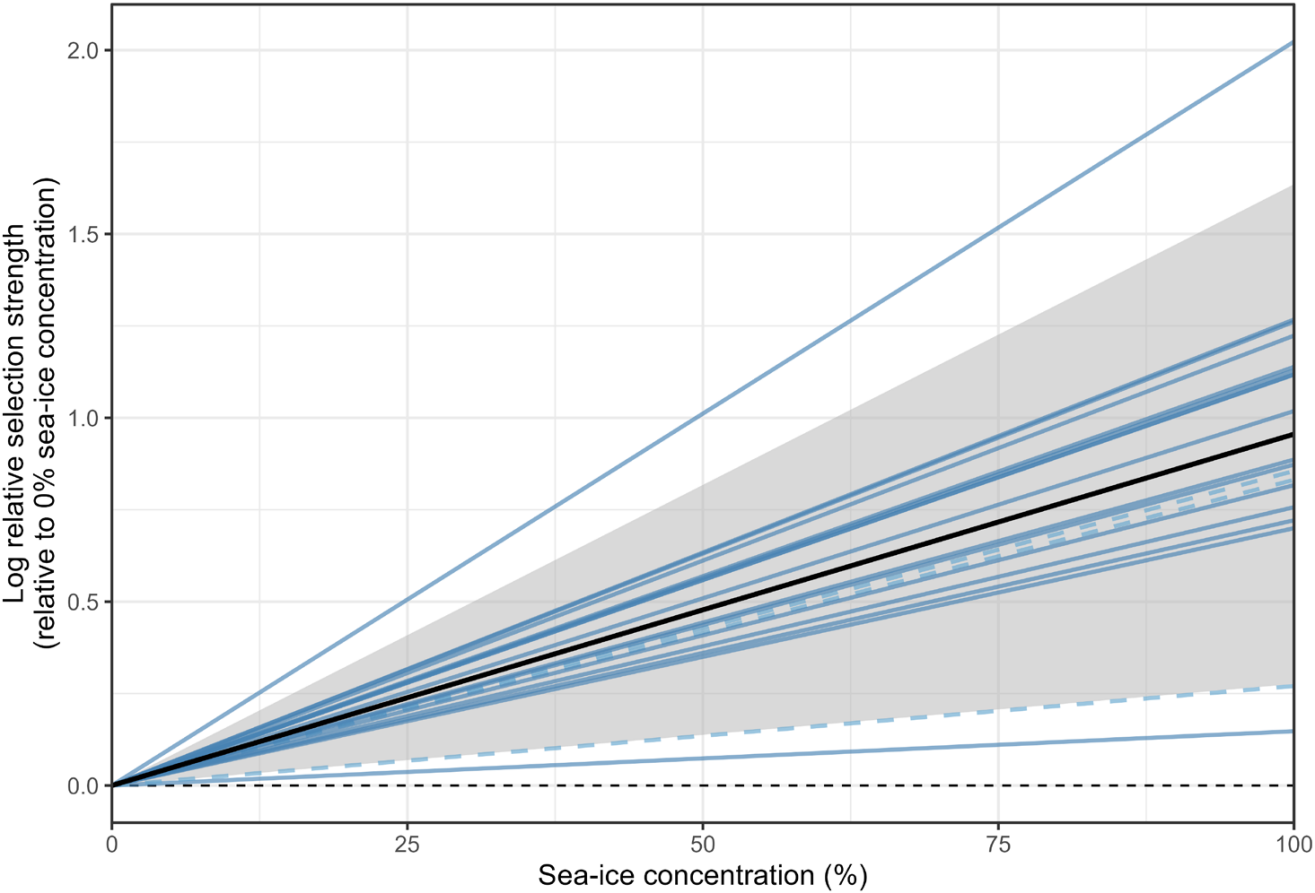
Model‐estimated log relative selection strength, log(RSS), for sea‐ice concentration by adult female polar bears in southeastern Hudson Bay and James Bay during the on‐ice period, 2012–2017. The x‐axis shows sea‐ice concentration from 0% to 100%. The black line shows the population‐level fixed‐effect estimate, and the gray ribbon shows the associated 95% confidence interval. Blue lines show individual‐level predictions, with dashed dark‐blue lines representing resident bears and dashed light‐blue lines representing non‐resident bears. The horizontal dashed line denotes log(RSS) = 0, indicating no difference in relative selection strength compared with 0% sea‐ice concentration.

Although our candidate set of models included formulations that allowed selection for sea‐ice concentration to vary temporally and between spatial clusters, the top‐ranking model indicated that overall bears consistently selected areas with high sea‐ice concentration relative to what was available throughout the on‐ice period. Population‐level relative selection strength remained positive during the winter, when sea‐ice concentration was near‐uniform, and during both freeze‐up and breakup, when sea‐ice conditions were more variable (Figure 4). Thus, bears continued to select areas with high sea‐ice concentration despite seasonal changes in availability, although the functional response term provided only weak evidence that the strength of selection varied with mean sea‐ice concentration among available locations within matched choice sets. Selection for sea‐ice concentration also varied among individuals (Figure 5), indicating that bears differed in the strength of selection for sea‐ice concentration despite a consistent positive population‐level effect.

**FIGURE 6:**
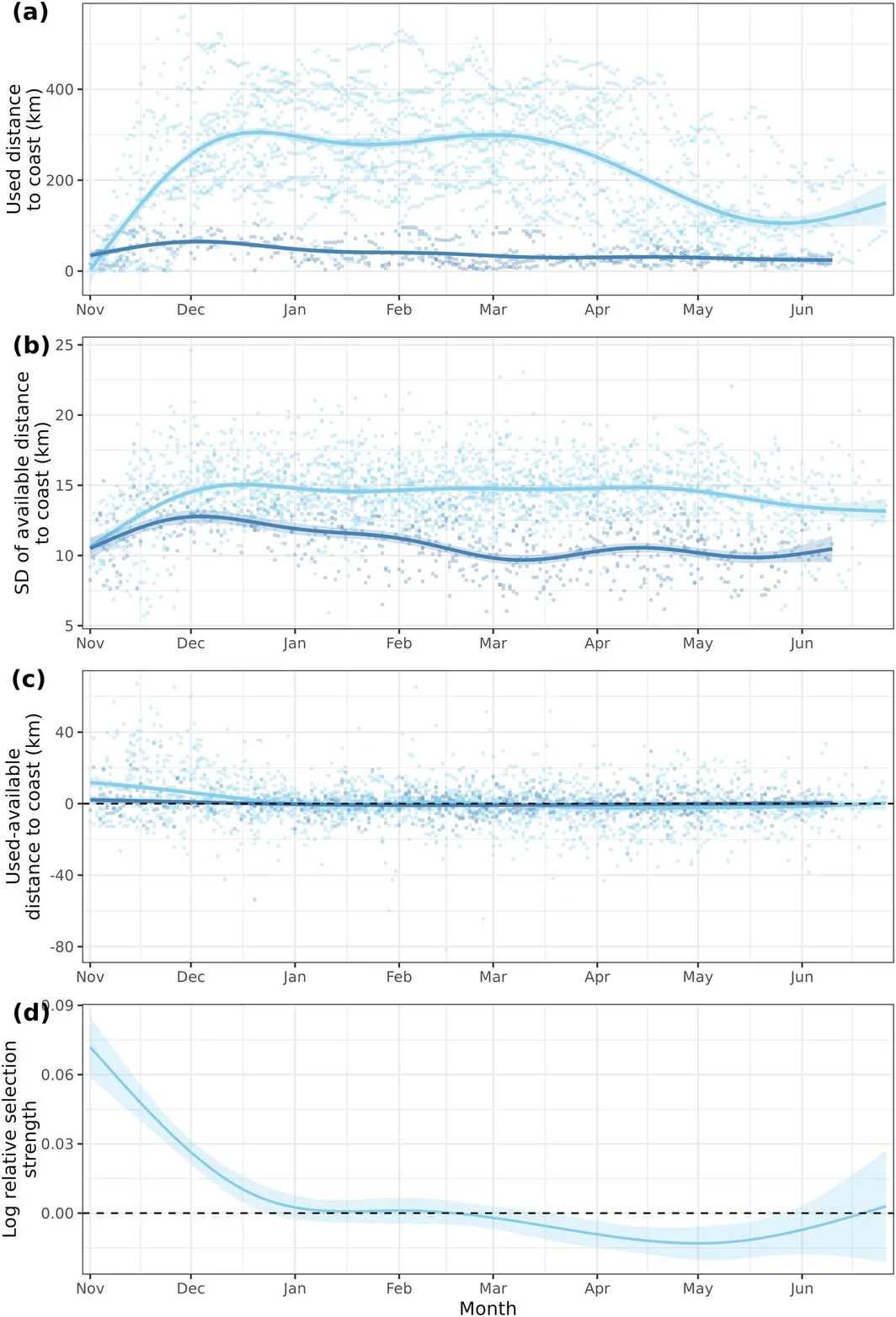
Seasonal patterns in distance‐to‐coast use, availability, and selection by polar bears in southeastern Hudson Bay and James Bay. (a) Distance to the coast at used locations. (b) Within‐stratum variation in available distance to coast, measured as the standard deviation among available locations in each used‐available choice set. (c) Difference between used and available distance to coast, calculated as used minus mean available distance within each stratum. Positive values indicate use of locations farther from the coast than was locally available, whereas negative values indicate use of locations closer to the coast. (d) Model‐estimated log relative selection strength, log(RSS), for a 1‐km increase in distance to coast among non‐resident bears; values above zero indicate selection for locations farther from the coast, whereas values below zero indicate selection for locations closer to the coast. Points in panels a–c show observed values, and lines show generalized additive smooths with 95% confidence intervals. Colors in panels a–c distinguish resident (dark blue) and non‐resident (light blue) bears.

The top‐ranking model indicated that relative selection strength for distance to the coast varied throughout the on‐ice period for non‐resident bears, with bears selecting areas farther from the coast during November to mid‐December and again in late June, although the magnitude of selection during June was weaker with greater uncertainty (Figure 6). During April to mid‐May, bears selected areas closer to the coast relative to what was available. Distance to the coast was not included for resident bears because it was highly correlated with ocean depth.

Ocean depth also influenced habitat selection, but its effect differed between resident and non‐resident bears. For non‐resident bears, relative selection strength varied over the on‐ice period (Figure S3) and among individuals (Figure S4). Selection for depth was weak through much of the season but increased later during the on‐ice period, with non‐resident bears selecting deeper areas by late May–June. Among resident bears, the estimated effect of ocean depth was negative but uncertain, suggesting weak evidence of selection for shallower areas relative to what was available.

Repeated withheld‐strata cross‐validation indicated the final model had limited out‐of‐sample discriminatory performance (*r*_*s*_ = 0.47; 95% CI = 0.32–0.58), with used locations not consistently assigned high relative choice probabilities. We suggest this result should be interpreted cautiously, however, as the test evaluates fine‐scale ranking within matched choice sets, which is difficult when used and available locations have similar covariate values. Limited contrast among used and available points was likely most pronounced during winter, when sea‐ice concentration in particular remained high and relatively uniform (Figure 4C), and may have been further compounded by the coarse spatial resolution of the data. Lastly, because the model was developed primarily for inference rather than prediction (Shmueli 2010; Tredennick et al. 2021), weak discriminatory performance does not invalidate the estimated selection patterns, but instead indicates limited ability to predict fine‐scale choices in withheld data.

## 4 Discussion

Intrapopulation variation in space use can lead to divergent space‐use patterns, often reflecting local environmental conditions to which individuals are adapted. However, changes in habitat due to ongoing climate change may disrupt these patterns, with possible consequences for genetic diversity and long‐term persistence (Tynan and DeMaster 1997; Pauls et al. 2013; Laidre et al. 2018; Peeters et al. 2020; Maduna et al. 2021). Our study examined polar bear spatial ecology at the southernmost extent of their global range, where individuals exhibited divergent patterns of broad‐scale space use. Some bears remained within relatively small, localized areas in southern James Bay, whereas others occupied larger ranges extending north of the bay. Together with previous genetic evidence, these movement patterns suggest that southern James Bay bears represent a spatially, behaviorally, and genetically structured part of the broader SH subpopulation. Despite differences in utilization distributions, individuals exhibited strong fidelity to James Bay and consistently selected areas with high sea ice concentration, highlighting the importance of this habitat feature even in a region where sea ice phenology has become increasingly variable (Durner et al. 2009; Stern and Laidre 2016). These results highlight the ecological and conservation importance of James Bay, particularly for bears that remain in the southern portion of the bay throughout the on‐ice period. As sea‐ice loss continues, reduced availability of high‐concentration ice may alter movement patterns and gene flow, potentially eroding the genetic distinctiveness of resident southern James Bay bears and/or increasing their risk of local decline.

### 4.1 Kernel Density Estimation and Spatial Clustering

Animals adapt their movements and range use in response to environmental heterogeneity, allowing them to track resources and reduce the influence of variable environments on life‐history processes (Ferguson et al. 1999; Nathan et al. 2008; Mauritzen et al. 2001; Horne et al. 2020). The large utilization distributions of polar bears, which are several orders of magnitude larger than similarly sized terrestrial carnivores, likely stem from bears modifying their movements in response to variation in sea‐ice conditions that influence the distribution of their prey (Ferguson et al. 1999). Consequently, bears inhabiting areas where sea‐ice conditions vary substantially within and among years are generally expected to have larger utilization distributions, as dynamic ice conditions alter the spatial and temporal availability of seals (Smith and Stirling 1978; Ferguson et al. 1999; Mauritzen et al. 2001). Despite occupying a region where sea‐ice phenology is highly variable, polar bears in southeastern Hudson Bay and James Bay had among the smallest mean utilization distributions reported for the species (Ferguson et al. 1999; Mauritzen et al. 2001; Auger‐Méthé et al. 2016; Gupta et al. 2022; Gough 2024). This may reflect a high degree of spatial fidelity to a relatively small area of high‐quality habitat, as bears consistently selected areas with high sea‐ice concentration, even during periods of overall low availability. Such behavior may allow individuals to remain within localized areas while still accessing sufficient foraging opportunities, representing an alternative space‐use strategy in a dynamic and increasingly unpredictable sea‐ice environment.

Polar bears in southeastern Hudson Bay and James Bay exhibited individual variation in both utilization distribution size and extent, adopting one of two general space‐use strategies. Three bears remained in James Bay between Akimiski Island and Charlton Island, with a pooled 95% utilization distribution of 15,323 km^2^. The remaining 12 bears had larger utilization distributions that encompassed areas surrounding the Belcher Islands, extending as far north as the Ottawa Islands, with a pooled 95% UD of 89,632 km^2^. Among other species of large mammals, including the closely related grizzly bear ( *U. arctos* ), intrapopulation variation in utilization distribution size and extent has been linked to density‐dependent factors, including intraspecific competition and variation in access to resources (McLoughlin et al. 2000; Kjellander et al. 2004; Mitchell and Powell 2012; Vander Wal et al. 2014). These mechanisms are difficult to evaluate here because prey availability and bear density during the on‐ice period are unknown; however, polar bears generally show limited evidence of habitat segregation according to competitive ability (Pilfold et al. 2014). Instead, the observed differences in space use may reflect individual experience or fidelity to familiar areas, a behavior well documented among adult female polar bears returning to maternal denning habitat (Ramsay and Stirling 1990; Amstrup and Gardner 1994; Zeyl et al. 2010; Klappstein, McGeachy, et al. 2024). Although evidence for on‐ice fidelity appears spatially variable (Mauritzen et al. 2001; Brun et al. 2021; Jodouin et al. 2025), individuals may return to areas where they have previously encountered reliable resources, resulting in distinct space‐use patterns among bears within the same population (Stirling et al. 2004; Börger et al. 2008; Van Moorter et al. 2009; Cherry et al. 2013; Riotte‐Lambert et al. 2015).

The degree of utilization distribution overlap between resident and non‐resident bears increased slightly during late winter, which, based on our seasonal delineation, encompassed the peak mating period in April–May (Ramsay and Stirling 1986; Stirling and Derocher 1993). However, the magnitude of overlap during both early and late winter remained near 0. Overlap estimates may be biased high for utilization distributions with very little overlap, meaning if the true overlap is 0, a few proximate locations may still yield a non‐zero estimate (Fieberg and Kochanny 2005). Therefore, our results indicate there was little or no overlap in seasonal utilization distributions between resident and non‐resident bears, including during the mating season, providing additional evidence that divergent space‐use strategies among polar bears in James Bay may reflect underlying ecological or behavioral differences that have contributed to previously documented intrapopulation genetic differentiation (Crompton et al. 2008; Obbard and Middel 2012; Peacock et al. 2015; Viengkone et al. 2018). Importantly, our study included only adult females. Although evidence suggests that adult male and female polar bears have broadly similar patterns of space use, despite differences in movement characteristics (e.g., path tortuosity) and breeding area range size (Amstrup et al. 2001; Laidre et al. 2013; Wilson et al. 2022), dispersal rates and the degree of utilization distribution overlap among other sex and age classes in SH, and James Bay specifically, remain unknown and warrant investigation.

### 4.2 On‐Ice Habitat Selection

Throughout the on‐ice period, polar bears in southeastern SH consistently selected areas with high sea‐ice concentration, including during freeze‐up and breakup, when sea‐ice conditions were more variable, and during winter, when conditions remained relatively uniform. Similar to results presented in Klappstein, McGeachy, et al. (2024); Klappstein, Michelot, et al. (2024), the strength of selection differed among individuals, but all bears exhibited the same general pattern. Areas with high concentrations of stable, first‐year ice provide an ideal environment for seals to rest, breed, and maintain breathing holes, thereby making them more accessible to polar bears (Smith 1980; Smith and Lydersen 1991; Kingsley and Stirling 1991; Kovacs et al. 2011). In addition to supporting higher prey densities, areas of high sea‐ice concentration also provide a stable platform for polar bears to rest and travel, reducing energetic costs associated with swimming and movement over open water or fragmented ice (Mauritzen et al. 2003; Durner et al. 2009; Pilfold et al. 2017). Collectively, our results indicate that sea‐ice concentration remains a critical determinant of habitat selection for polar bears across southeastern SH throughout the on‐ice period. As climate‐change‐mediated warming continues to shorten the ice‐covered period and increase the prevalence of fragmented or marginal ice, the availability of high‐quality habitat is expected to decline. Given the consistent selection for high‐concentration ice, continued reductions in stable sea‐ice habitat may alter movement patterns and reduce access to prey, likely increasing nutritional stress.

For non‐resident bears, distance to the Ontario coast varied seasonally and was the most important covariate in terms of relative magnitude of selection, similar to results obtained in neighboring Western Hudson Bay (McCall et al. 2016). During November to mid‐December, bears selected areas farther from the coast, likely following the advancing ice edge where newly formed sea ice may provide early opportunities to hunt seals (Ferguson et al. 2000; Durner et al. 2009; McCall et al. 2016; Middel and Obbard 2024). After freeze‐up, relative selection strength associated with distance to the coast remained near 1, suggesting that once bears had moved offshore, movements within those offshore areas were not strongly influenced by local variation in proximity to the coast. By April and May, however, non‐resident bears selected areas closer to shore, coinciding with ringed seal pupping and nursing season, when adult females and newborn pups are concentrated in nearshore fast‐ice and predation by polar bears increases (Stirling and Archibald 1977; Smith 1980; Parks et al. 2006; Chambellant et al. 2012). During late spring and breakup, bears again appeared to select areas farther from shore, likely to prolong access to seals on remnant sea ice (Stirling et al. 1999; Obbard et al. 2015); however, estimates of relative selection strength overlapped 1, suggesting this pattern was weaker and more uncertain than during freeze‐up.

Ocean depth appeared to have limited influence on habitat selection in our study, but differed between resident and non‐resident bears. For non‐resident bears, selection for ocean depth was weak through much of the on‐ice period but increased during late spring when bears selected deeper areas during late May to June. Among resident bears, the estimated effect of depth was negative but highly uncertain. In other regions of the Arctic, where ocean depths are more varied, polar bears tend to select shallower areas over the continental shelf where ocean productivity is highest (Derocher et al. 2004; Durner et al. 2009; Wilson et al. 2014). The entire SH area is comparatively shallow, averaging 86 m (SD: 58 m), and only 60 m (SD: 23 m) in James Bay. Consequently, depth may be a weaker proxy for productivity gradients in SH than in regions with more heterogeneous bathymetry. Instead, the late‐season selection for deeper areas by non‐resident bears may reflect use of offshore habitats where remnant sea ice persists later into breakup, whereas the absence of strong selection among resident bears may reflect the uniformly shallow depth of southern James Bay.

Our analysis did not explicitly examine polar bear selection for prey resources. Rather, we focused on factors related to prey accessibility in relation to sea‐ice dynamics due to a lack of comprehensive data on prey densities and distribution in SH. Additionally, we pooled data from all the bears in our study to maximize sample sizes and inferential power, ignoring possible differences in age and reproductive status that may influence patterns of selection, particularly for pregnant females and those with cubs‐of‐the‐year (Stirling et al. 1993; Mauritzen et al. 2003; Freitas et al. 2012; McCall et al. 2016). Future research should account for these factors, prioritizing collection of prey density and distribution information, as well as larger sample sizes to better analyze the influence of demographic characteristics on selection.

### 4.3 Management Implications

The observed spatial segregation between resident and non‐resident bears in James Bay suggests that persistent intrapopulation differences in space‐use contributed to the distinct genetic structure previously identified in southern James Bay bears (Crompton et al. 2008; Obbard and Middel 2012; Peacock et al. 2015; Viengkone et al. 2018). Studies of polar bears captured in James Bay between 1997 and 2012 documented genetic differences among individuals that migrated to Hudson Bay during the on‐ice period and those that remained in southern James Bay (Crompton et al. 2008; Peacock et al. 2015; Malenfant et al. 2016; Viengkone et al. 2018). Bears that moved northward appear to have interbred with individuals from neighboring Western Hudson Bay and Foxe Basin, consistent with a metapopulation‐like structure in which semi‐discrete groups remain connected through seasonal movements and range overlap, facilitating demographic exchange and gene flow (Hanski and Gilpin 1991; Hanski 1998). In contrast, bears that remained in southern James Bay formed a distinct genetic cluster. Our results demonstrate that this spatial segregation persisted at least through 2017, with some bears remaining in southern James Bay during the entire on‐ice period, including during the breeding season, suggesting demographic and genetic connectivity has likely remained limited for this group.

Intrapopulation differences in space‐use have been documented in other polar bear subpopulations (Pagano et al. 2021; Mauritzen et al. 2001, 2002). In the Barents Sea, for example, a portion of the subpopulation remains in nearshore habitat throughout the Svalbard Archipelago, whereas the remainder of the subpopulation generally spends the summer on offshore pack ice in the marginal ice zone between Svalbard, Franz Josef Land, and Novaya Zemlya (Mauritzen et al. 2001, 2002; Maduna et al. 2021). Although sea‐ice loss appears to be reducing seasonal connectivity between these areas, “pelagic” bears still move near the Svalbard Archipelago during winter and spring, including during the breeding season, maintaining opportunities for interbreeding between the two groups (Maduna et al. 2021). James Bay bears appear to differ in this respect, as the restricted space‐use of resident bears documented here and in previous studies may preclude or limit exchange with bears in the broader Hudson Bay complex (Crompton et al. 2008; Peacock et al. 2015; Viengkone et al. 2018). In this sense, bears that remain in southern James Bay share some similarities with those in Southeast Greenland, where restricted movements, limited immigration, and distinct genetic structure contributed to their designation as a separate management unit (Laidre et al. 2022). The comparison is imperfect, however, as Southeast Greenland bears are geographically constrained by physical barriers that limit dispersal and immigration, whereas James Bay lacks comparable barriers and remains connected to Hudson Bay by continuous sea ice during the winter. Moreover, resident and non‐resident bears overlap extensively on land during the ice‐free period, when individuals return to the Ontario mainland or islands in James Bay (Obbard and Middel 2012).

Intrapopulation variation in space use among polar bears in James Bay and other subpopulations illustrates a broader management challenge, as spatial structuring can occur both within and among subpopulations, and the boundaries of biologically meaningful units may therefore not always align with existing delineations which often are developed through a mix of biological and sociopolitical drivers. For instance, caribou ( *Rangifer tarandus* ) occupy broad, continuous distributions comprised of relatively discrete subunits separated by seasonal movements, behavioral differences, or temporally variable barriers (Poole et al. 2010; Mallory and Boyce 2018; Peeters et al. 2020; Hughes et al. 2025). Consequently, they have become a useful example of how conservation units can be defined using multiple lines of evidence, including genetics and space use (Yannic et al. 2016; Jenkins et al. 2018; Hughes et al. 2025). Although no single set of criteria provides a universal basis for defining conservation units, and different approaches can yield different results (see Yannic et al. 2016), identifying biologically meaningful units remains critical because failing to account for spatially structured groups can obscure important differences in connectivity, demography, and vulnerability to environmental change and anthropogenic exploitation.

The pooled 95% utilization distributions of resident and non‐resident bears were entirely within SH, concentrated in the eastern and southern portions of the subpopulation boundary. Bears captured during previous studies along the northern Ontario coast showed a greater tendency to move throughout central and western Hudson Bay, resulting in utilization distributions that extended into neighboring subpopulations (Obbard and Middel 2012; Viengkone et al. 2018). Bears that remain in central Hudson Bay are less vulnerable to harvest, as this area is essentially inaccessible to hunters (Jonkel et al. 1976; Stirling et al. 2004; Obbard et al. 2007). In contrast, bears that remain in James Bay and southeastern Hudson Bay are more vulnerable to harvest due to the areas' proximity to communities in Nunavut, Ontario, and Quebec. Based on tag returns from bears marked during previous mark‐recapture studies, the majority of bears reported in the Nunavut and Quebec harvests were originally tagged east of longitude 88° W on the Ontario coast of Hudson Bay and James Bay or on islands in James Bay, suggesting a higher hunting pressure and mortality rate for bears in these regions (Jonkel et al. 1976; Obbard et al. 2007). Although both resident and non‐resident bears may be more vulnerable to harvest, mortality among resident bears likely poses a greater conservation concern because it could reduce unique genetic variation within SH and may be less readily offset by compensatory immigration.

## 5 Conclusions

Our results, along with previous research, indicate polar bears exhibit persistent preferences for specific habitat characteristics, particularly areas with high sea‐ice concentration, as its availability has changed over recent decades (Durner et al. 2009, 2019; Wilson et al. 2016). Elsewhere in the Arctic, enduring habitat preferences may be contributing to divergent range use strategies, as polar bears within the same subpopulation move to different areas to maintain access to disparate high‐quality habitat (Mauritzen et al. 2001, 2002; Pagano et al. 2021), resulting in increased genetic isolation in at least one subpopulation (Maduna et al. 2021). For polar bears in James Bay, however, the divergent space‐use strategies observed here and inferred in previous studies may ultimately converge as sea ice loss continues. Bears that currently remain in southern James Bay during the on‐ice period may be forced to move north toward more persistent ice, resulting in greater utilization distribution overlap and loss of genetic diversity within the SH subpopulation as bears more readily interbreed. The potential loss of intrapopulation genetic diversity is of particular concern, as some of these bears appear to be locally adapted to conditions in southern James Bay—a loss that would diminish the adaptive resilience of polar bears within SH and regionally, constituting a significant conservation failure.

Although resident bears in southern James Bay are not geographically constrained by physical barriers, as is the case in Southeast Greenland, our results, together with previous genetic evidence, suggest they represent a spatially, behaviorally, and genetically structured component of the broader SH subpopulation. Their limited distribution, restricted overlap with other bears, and genetic differentiation indicate some degree of demographic isolation that likely renders them more vulnerable to habitat degradation and both natural and harvest‐related mortality. We therefore suggest that southern James Bay bears warrant consideration as a distinct conservation unit within SH, and that population monitoring and harvest risk assessments explicitly account for this distinct group. However, additional research to refine our understanding of the level of distinctness and the drivers of this potential demographic separation is needed. Continued monitoring of kinship and gene flow would also help determine whether sea‐ice loss leads to increased admixture between southern James Bay bears and other SH bears, eroding their genetic distinctiveness.

Polar bears exhibit physiological and behavioral plasticity that may enable them to adapt to changing conditions in the short‐ to medium‐term (Stirling et al. 1999; Pagano et al. 2012, 2024; Galicia et al. 2021). However, as sea ice continues to decline over the long term, polar bears will likely be forced to occupy increasingly marginal habitats, which is expected to result in continued declines in body condition, recruitment, and ultimately population abundance, as has been documented in other areas (Regehr et al. 2007, 2010; Stirling and Derocher 2012; Molnár et al. 2020). Recent projections suggest that if trends in Arctic warming continue unabated, polar bears in SH are likely to experience significant demographic decline, including reduced survival and cub recruitment, and may ultimately become extirpated by the turn of the century (Molnár et al. 2020; Stroeve et al. 2024). Therefore, continued monitoring of polar bears in SH, and James Bay in particular, where the open‐water season is among the longest across the species' global range, is critical for tracking these changes and informing possible corrective or mitigative management efforts.

## Author Contributions

**Martyn E. Obbard:** data curation, writing – review and editing, project administration, resources, validation, funding acquisition, conceptualization. **Tyler R. Ross:** writing – original draft, investigation, conceptualization, visualization, methodology, validation, formal analysis, data curation. **Kevin R. Middel:** writing – review and editing, validation, project administration, resources. **Gregory W. Thiemann:** investigation, funding acquisition, writing – review and editing, validation, supervision, resources, conceptualization. **Joseph M. Northrup:** funding acquisition, writing – review and editing, validation, methodology, formal analysis, supervision, resources, project administration.

## Funding

Funding was provided by the Ontario Ministry of Natural Resources' Wildlife Research and Monitoring Section and Species at Risk Research Fund for Ontario (SARRFO), Polar Bears International, Toronto Zoo's Endangered Species Fund, Wildlife Conservation Society Canada, Canadian Wildlife Federation, Born Free Foundation, and the NSERC Discovery Grants Program.

## Conflicts of Interest

The authors declare no conflicts of interest.

## Supporting information


**Figure S1:** Hierarchical clustering of Bhattacharyya distances (BD) among utilization distributions (UDs) for 15 adult female polar bears captured and GPS‐collared in James Bay between 2012 and 2015. Mean silhouette widths indicated bears were optimally clustered into two distinct groups, here denoted as the Northern James Bay cluster (non‐resident bears; light blue) and Southern James Bay cluster (resident bears; dark blue).
**Figure S2:** Empirical distributions of step lengths (km) and turn angle (radians) for used steps by resident (dark blue solid line) and non‐resident (light blue dashed line) adult female polar bears in southeastern Hudson Bay and James Bay, 2012–2017.
**Figure S3:** Seasonal patterns in ocean depth use, availability, and selection by polar bears in southeastern Hudson Bay and James Bay. (A) Ocean depth at used locations. (B) Within‐stratum variation in available ocean depth, measured as the standard deviation among available locations in each used‐available choice set. (C) Difference between used and available ocean depth, calculated as used minus mean available depth within each stratum. Positive values indicate use of deeper water than was locally available, whereas negative values indicate use of shallower water. (D) Model‐estimated log relative selection strength, log(RSS), for a 1 m increase in ocean depth; values above zero indicate selection for deeper water, whereas values below zero indicate selection for shallower water. Points in panels A–C show observed values and lines show generalized additive smooths with 95% confidence intervals. Colors distinguish resident (dark blue) and non‐resident (light blue) bears. Based on results of model selection, the top‐ranking model indicated the effect of ocean depth differed between resident and non‐resident bears: the resident depth effect was modeled as a fixed slope, whereas the non‐resident depth effect varied over time, so the resident log(RSS) is constant through time and the non‐resident log(RSS) changes across the on‐ice period.
**Figure S4:** Model‐estimated log relative selection strength, log(RSS), for ocean depth by adult female non‐resident polar bears in southeastern Hudson Bay during the on‐ice period, 2012–2017. The x‐axis shows ocean depth in meters. The black line shows the population‐level fixed effect estimate, and the gray ribbon shows the associated 95% confidence interval. Blue lines show individual‐level predictions for non‐resident bears. The horizontal dashed line denotes log(RSS) = 0, indicating no difference in relative selection strength compared with 0 m depth. Estimates are shown for the median season day of May–June (May 27), when non‐resident bears generally selected deeper water.
**Figure S5:** Used‐habitat calibration plots for the top‐ranked step selection function model evaluating habitat selection by adult female polar bears in southeastern Hudson Bay and James Bay. Plots compare the observed distribution of covariate values at used locations with the distribution predicted by the fitted model. Solid black lines show observed used locations, gray bands show model‐predicted used locations, and red dashed lines show available locations within used‐available matched choice sets. Greater overlap between the black line and gray bands indicates stronger model calibration (Fieberg et al. 2018). Distance to the coast was not evaluated for resident bears because it exhibited relatively little variation and was highly correlated with ocean depth. Therefore, it was omitted from the final model for resident bears.
**Table S1:** Model‐selection results for candidate step selection function (SSF) models evaluating selection for sea‐ice concentration, ocean depth, distance to coast, temporal variation in selection, functional responses, and individual variation among adult female polar bears in southeastern Hudson Bay and James Bay, 2012–2017. Candidate models were developed using a two‐stage approach, in which a constrained global model1 was first used to identify supported temporal smooths and individual‐level random effects, followed by comparison of a reduced set of biologically plausible models that differed in fixed‐effect environmental covariates. Models are ranked by corrected conditional AIC. Movement terms included step length, log‐transformed step length (log(step.length)), and the cosine of turn angle (cos(turn.angle)). Habitat covariates included sea‐ice concentration (conc), ocean depth (depth), and distance to the nearest coastline (dist). Group‐specific terms are denoted by .res for resident bears and .nonres for non‐resident bears. Functional response terms are denoted by fr.ice and represent interactions with stratum specific sea‐ice availability. Smooth terms, s(season.day, covariate), represent continuous temporal variation in selection for the corresponding covariate across the on‐ice period, where season.day is the continuous day of the on‐ice season. Random‐slope terms (covariate | individual), represent individual‐level variation in selection. Columns show model formulae, estimated degrees of freedom (edf), AIC, difference in AIC relative to the top‐ranking model (ΔAIC), Akaike weight (w), and log‐likelihood (logLik).

## Data Availability

Data supporting this research are sensitive and not publicly available. Polar bear spatial data are owned by the Ontario government and incorporated into the Ontario Natural Heritage Information Centre (NHIC). A license agreement may be requested to access sensitive information by visiting the NHIC website (https://www.ontario.ca/page/natural‐heritage‐information‐centre) or by contacting nhicrequests@ontario.ca. Requests for data require an application for a Sensitive Data Use License.
