# Supplementary material for: Habitat Selection and Intrapopulation Spatial Segregation in an Arctic Top Predator at the Southern Limit of Its Range
Source: Glob Chang Biol. 2026 Sep 5;32(9):e71024. doi: 10.1111/gcb.71024 (PMC13545663; doi:10.1111/gcb.71024)
Supplement: Supplementary file 1 [file GCB-32-e71024-s001.pdf]

SUPPORTING INFORMATION | HABITAT SELECTION AND INTRAPOPULATION SPATIAL SEGREGATION IN AN ARCTIC TOP PREDATOR AT THE SOUTHERN LIMIT OF ITS RANGE

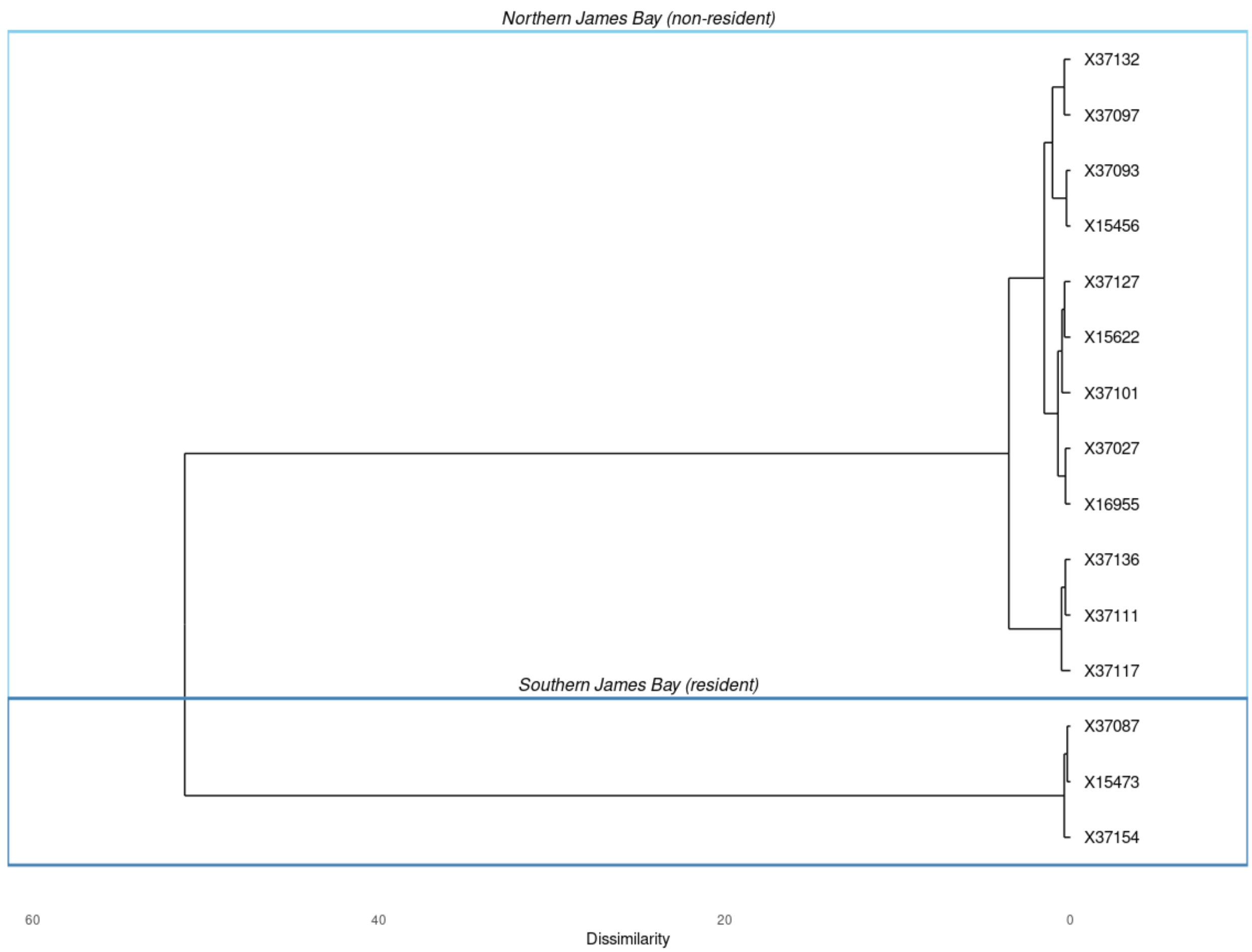


Figure S1. Hierarchical clustering of Bhattacharyya distances (BD) among utilization distributions (UDs) for 15 adult female polar bears captured and GPS-collared in James Bay between 2012 and 2015. Mean silhouette widths indicated bears were optimally clustered into two distinct groups, here denoted as the Northern James Bay cluster (non-resident bears; light blue) and Southern James Bay cluster (resident bears; dark blue).

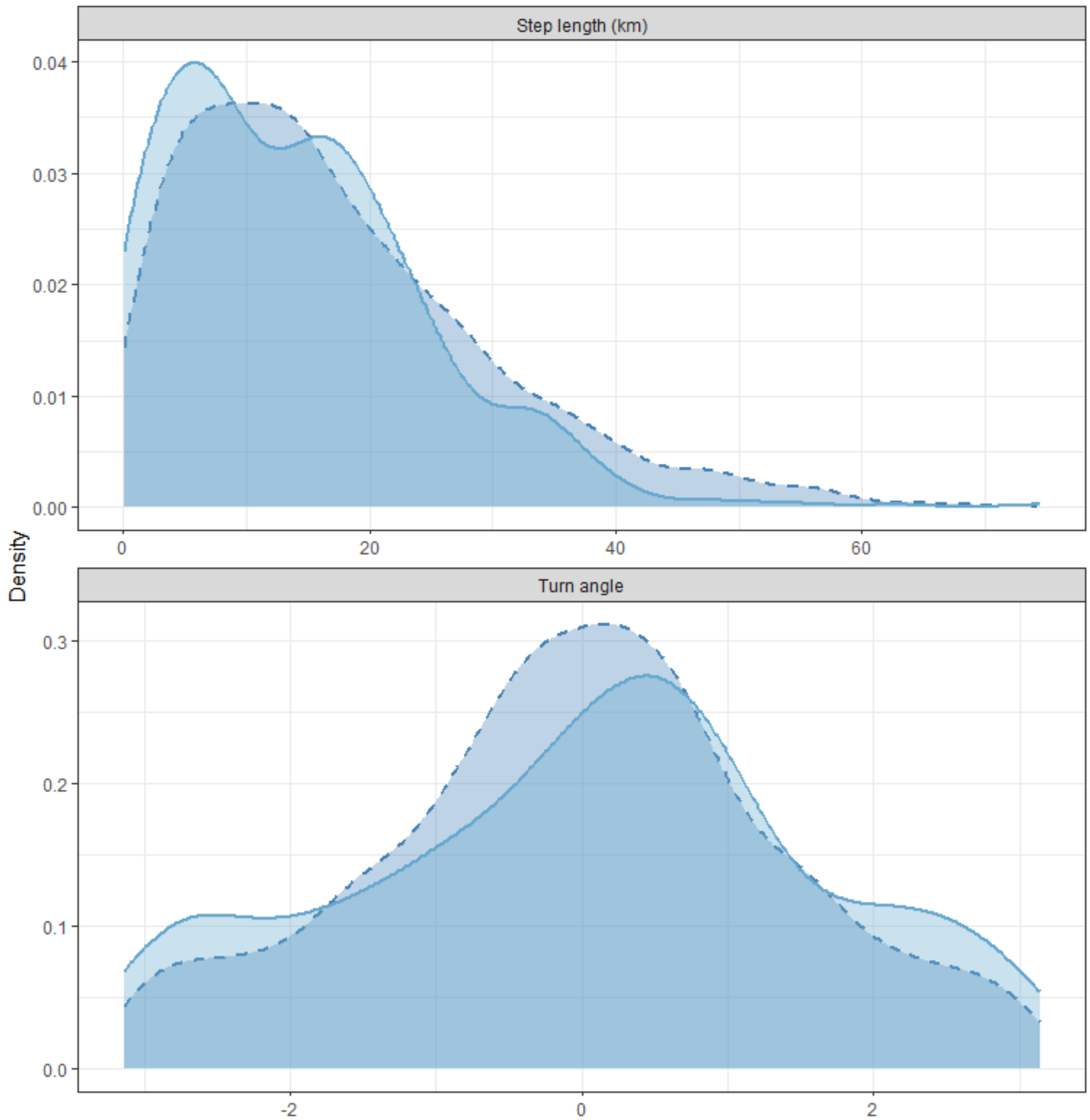


Figure S2. Empirical distributions of step lengths (km) and turn angle (radians) for used steps by resident (dark blue solid line) and non-resident (light blue dashed line) adult female polar bears in southeastern Hudson Bay and James Bay, 2012-2017.

Table S1. Model-selection results for candidate step selection function (SSF) models evaluating selection for sea-ice concentration, ocean depth, distance to coast, temporal variation in selection, functional responses, and individual variation among adult female polar bears in southeastern Hudson Bay and James Bay, 2012-2017. Candidate models were developed using a two-stage approach, in which a constrained global model[1] was first used to identify supported temporal smooths and individual-level random effects, followed by comparison of a reduced set of biologically plausible models that differed in fixed-effect environmental covariates. Models are ranked by corrected conditional AIC. Movement terms included *step length*, log-transformed step length (*log(step.length)*), and the cosine of turn angle (*cos(turn.angle)*). Habitat covariates included sea-ice concentration (*conc*), ocean depth (*depth*), and distance to the nearest coastline (*dist*). Group-specific terms are denoted by *.res* for resident bears and *.nonres* for non-resident bears. Functional response terms are denoted by *fr.ice* and represent interactions with stratum-specific sea-ice availability. Smooth terms, *s(season.day, covariate)*, represent continuous temporal variation in selection for the corresponding covariate across the on-ice period, where *season.day* is the continuous day of the on-ice season. Random-slope terms, (*covariate* | *individual*), represent individual-level variation in selection. Columns show model formulae, estimated degrees of freedom (edf), AIC, difference in AIC relative to the top-ranking model (ΔAIC), Akaike weight (w), and log-likelihood (logLik).

| Model structure | edf | AIC | ΔAIC | w | logLik |
|---|---|---|---|---|---|
| step.length + log(step.length) + cos(turn.angle) + conc + depth.nonres + depth.res + dist.nonres + fr.ice + s(season.day, depth.nonres) + s(season.day, dist.nonres) + (conc \| individual) + (depth.nonres \| individual) | 29.39 | 26717.86 | 0.00 | 0.54 | -13329.54 |
| step.length + log(step.length) + cos(turn.angle) + conc.nonres + conc.res + depth.nonres + depth.res + dist.nonres + fr.ice + s(season.day, depth.nonres) + s(season.day, dist.nonres) + (conc \| individual) + (depth.nonres \| individual) | 29.79 | 26718.45 | 0.59 | 0.40 | -13329.44 |

| | | | | | |
|---|---|---|---|---|---|
| step.length + log(step.length) + cos(turn.angle) + conc + depth.nonres + depth.res + dist.nonres + s(season.day, depth.nonres) + s(season.day, dist.nonres) + (conc \| individual) + (depth.nonres \| individual) | 31.31 | 26724.29 | 6.43 | 0.02 | -13330.84 |
| step.length + log(step.length) + cos(turn.angle) + conc + depth + dist + s(season.day, depth) + s(season.day, dist) + (conc \| individual) + (depth \| individual) | 27.90 | 26724.96 | 7.10 | 0.02 | -13334.58 |
| step.length + log(step.length) + cos(turn.angle) + conc + depth.nonres + dist.nonres + fr.ice + s(season.day, depth.nonres) + s(season.day, dist.nonres) + (conc \| individual) + (depth.nonres \| individual) | 31.51 | 26725.51 | 7.65 | 0.01 | -13331.24 |
| step.length + log(step.length) + cos(turn.angle) + conc + depth.nonres + dist.nonres + s(season.day, depth.nonres) + s(season.day, dist.nonres) + (conc \| individual) + (depth.nonres \| individual) | 30.40 | 26725.99 | 8.13 | 0.01 | -13332.60 |
| step.length + log(step.length) + cos(turn.angle) + conc + depth.nonres + dist + s(season.day, depth.nonres) + s(season.day, dist) + (conc \| individual) + (depth.nonres \| individual) | 29.67 | 26732.49 | 14.63 | <0.01 | -13336.57 |
| step.length + log(step.length) + cos(turn.angle) + conc + depth.nonres + depth.res + dist.nonres | 7.00 | 26869.02 | 151.16 | <0.01 | -13427.51 |
| step.length + log(step.length) + cos(turn.angle) + conc + depth + dist | 6.00 | 26872.31 | 154.45 | <0.01 | -13430.16 |

[1] The constrained global model: step.length + log(step.length) + cos(turn.angle) + conc.nonres + conc.res + depth.nonres + depth.res + dist.nonres + fr.ice + s(season.day, conc) + s(season.day, depth.nonres) + s(season.day, dist.nonres) + (conc | individual) + (depth.nonres | individual.nonres) + (dist.nonres | individual.nonres). The model was fit using the "double-penalty" approach (Marra & Wood 2011, Klappstein et al. 2024), which allowed weakly supported smooth and random slope terms to be penalized toward zero, effectively removing them from the model. Resident bears were not included in temporal smooth or individual-level random effects because there were only three individuals. Initial models with resident-specific terms resulted in model identifiability issues. This formulation therefore represents the maximally constrained global model used to identify supported temporal and individual-level terms. Those terms reduced to near zero (s(season.day, conc) and (dist.nonres | individual.nonres)) were not retained in the reduced candidate model set.

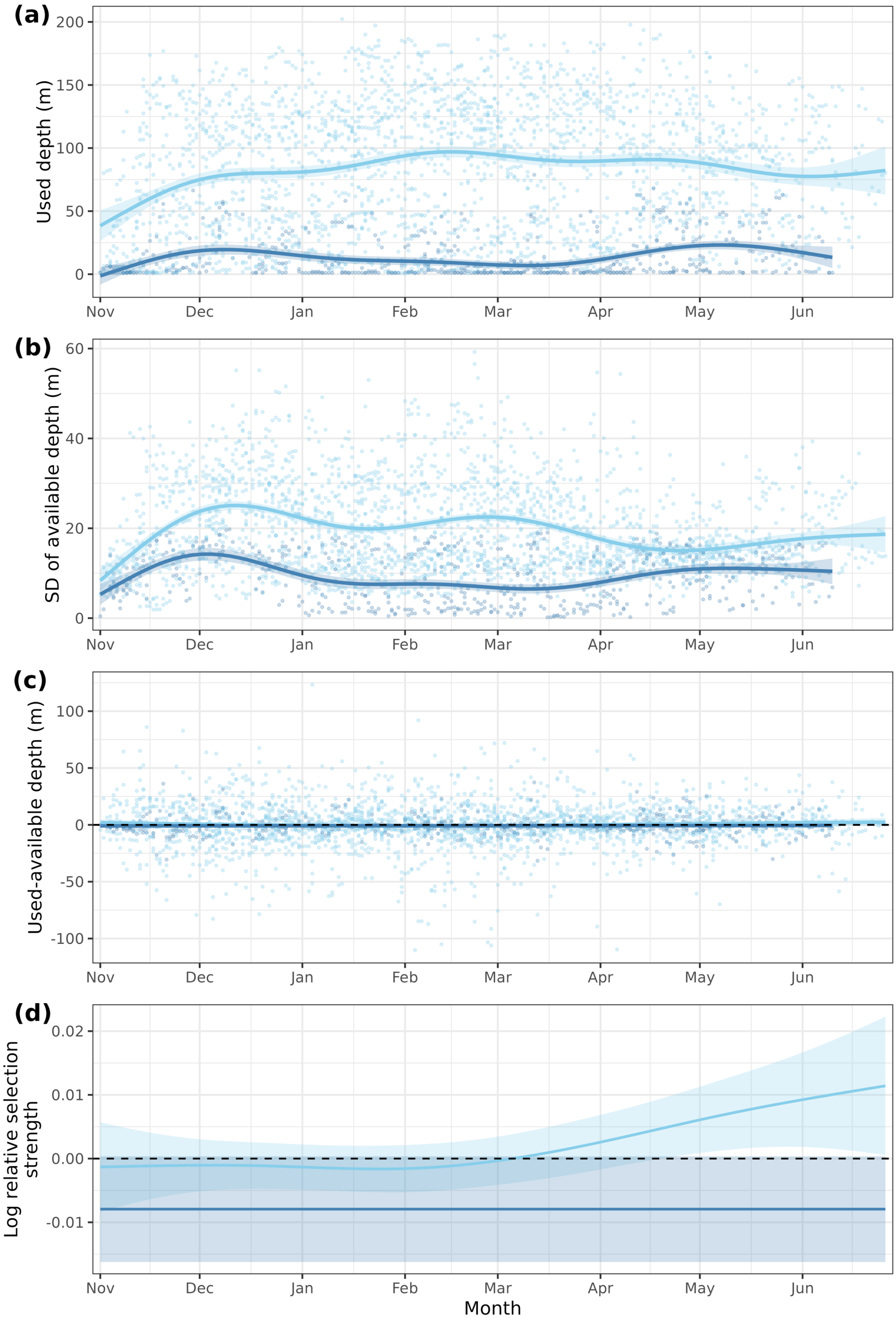

(a)
Used depth (m)
200
150
100
50
0
(b)
SD of available depth (m)
60
40
20
0
(c)
Used-available depth (m)
100
50
0
-50
-100
(d)
Log relative selection strength
0.02
0.01
0.00
-0.01
Nov
Dec
Jan
Feb
Mar
Apr
May
Jun
Month


1

Figure S3. Seasonal patterns in ocean depth use, availability, and selection by polar bears in southeastern Hudson Bay and James Bay. (A) Ocean depth at used locations. (B) Within-stratum variation in available ocean depth, measured as the standard deviation among available locations in each used-available choice set. (C) Difference between used and available ocean depth, calculated as used minus mean available depth within each stratum. Positive values indicate use of deeper water than was locally available, whereas negative values indicate use of shallower water. (D) Model-estimated log relative selection strength, log(RSS), for a 1 m increase in ocean depth; values above zero indicate selection for deeper water, whereas values below zero indicate selection for shallower water. Points in panels A–C show observed values and lines show generalized additive smooths with 95% confidence intervals. Colours distinguish resident (dark blue) and non-resident (light blue) bears. Based on results of model selection, the top-ranking model indicated the effect of ocean depth differed between resident and non-resident bears: the resident depth effect was modelled as a fixed slope, whereas the non-resident depth effect varied over time, so the resident log(RSS) is constant through time and the non-resident log(RSS) changes across the on-ice period.

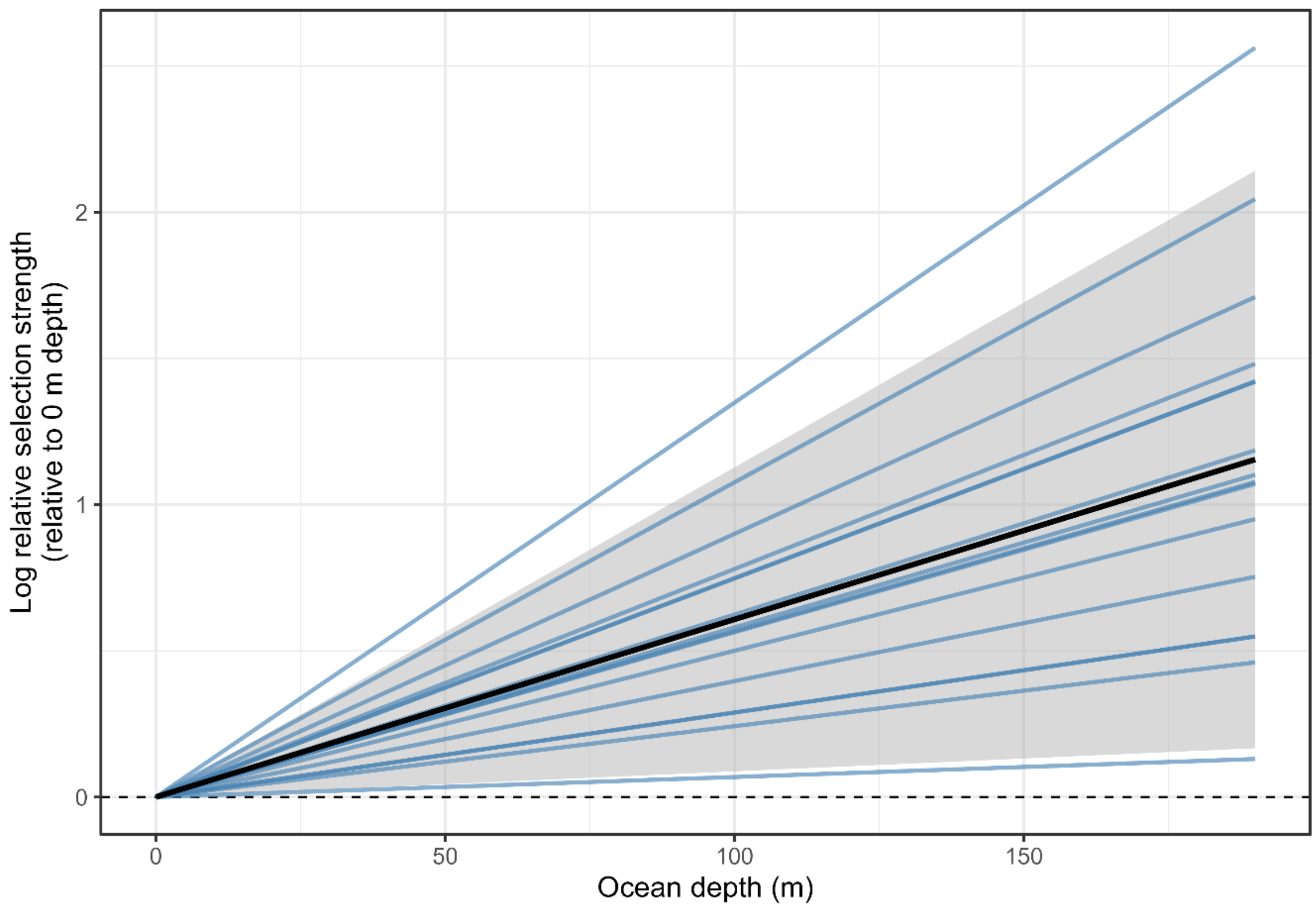


Figure S4. Model-estimated log relative selection strength, log(RSS), for ocean depth by adult female non-resident polar bears in southeastern Hudson Bay during the on-ice period, 2012-2017. The x-axis shows ocean depth in metres. The black line shows the population-level fixed-effect estimate, and the grey ribbon shows the associated 95% confidence interval. Blue lines show individual-level predictions for non-resident bears. The horizontal dashed line denotes log(RSS) = 0, indicating no difference in relative selection strength compared with 0 m depth. Estimates are shown for the median season day of May-June (May 27), when non-resident bears generally selected deeper water.

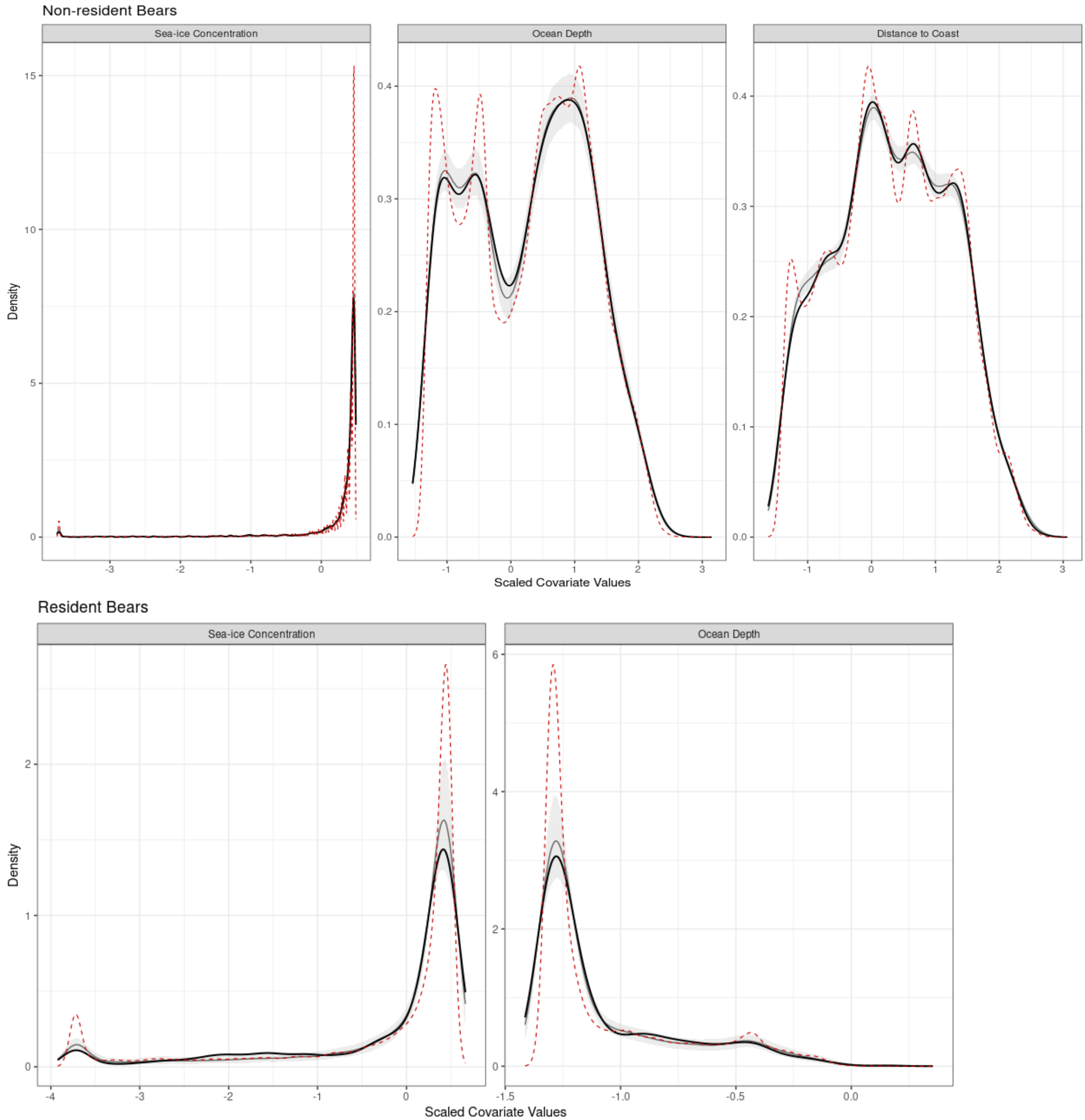


Figure S5. Used-habitat calibration plots for the top-ranked step selection function model evaluating habitat selection by adult female polar bears in southeastern Hudson Bay and James Bay. Plots compare the observed distribution of covariate values at used locations with the

distribution predicted by the fitted model. Solid black lines show observed used locations, grey bands show model-predicted used locations, and red dashed lines show available locations within used-available matched choice sets. Greater overlap between the black line and grey bands indicates stronger model calibration (Fieberg et al. 2018). Distance to the coast was not evaluated for resident bears because it exhibited relatively little variation and was highly correlated with ocean depth. Therefore, it was omitted from the final model for resident bears.
